# Comparison of the RECIST and PERCIST criteria in solid tumors: a pooled analysis and review

**DOI:** 10.18632/oncotarget.8425

**Published:** 2016-03-28

**Authors:** Seon Jeong Min, Hyun Joo Jang, Jung Han Kim

**Affiliations:** ^1^ Department of Radiology, Dongtan Sacred-Heart Hospital, Hallym University Medical Center, Hallym University College of Medicine, Hwasung 18450, Republic of Korea; ^2^ Division of Gastroenterology, Department of Internal Medicine, Dongtan Sacred-Heart Hospital, Hallym University Medical Center, Hallym University College of Medicine, Hwasung 18450, Republic of Korea; ^3^ Division of Hemato-Oncology, Department of Internal Medicine, Kangnam Sacred-Heart Hospital, Hallym University Medical Center, Hallym University College of Medicine, Seoul 07441, Republic of Korea

**Keywords:** RECIST 1.0, RECIST 1.1, PET, PERCIST, tumor response

## Abstract

The PET Response Criteria in Solid Tumors (PERCIST) is a new method for the quantitative assessment of metabolic changes in solid tumors. The assessments of tumor response between the RECIST and PERCIST have shown considerable difference in several studies. This pooled study was conducted to compare tumor response according to the two criteria in patients with solid tumors. We surveyed MEDLINE, EMBASE and PUBMED for articles with terms of the RECIST or PERCIST from 2009 and January 2016. There were six articles comparing the RECIST and PERCIST. A total of 268 patients were recruited; 81 with colorectal cancer, 60 with lung cancer, 48 with esophageal cancer, 28 with breast cancer, 14 with basal cell carcinoma, 12 with stomach cancer, 10 with head and neck cancer, and 16 with other rare cancers. The agreement of tumor response between the RECIST and PERCIST was moderate (k = 0.590). Of 268 patients, 101 (37.7%) showed discordance in the tumor responses between two criteria. When adopting the PERCIST, tumor response was upgraded in 85 patients and downgraded in 16. The estimated overall response rates were significantly different between two criteria (35.1% by RECIST vs. 54.1% by PERCIST, *P* < 0.0001). In conclusion, this pooled analysis demonstrates that the concordance of tumor responses between the RECIST and PERCIST criteria is not excellent. The PERCIST might be more suitable for assessing tumor response than the RECIST criteria.

## INTRODUCTION

To avoid continuing anti-cancer treatment with no efficacy, the accurate assessment of therapeutic response is essential. The Response Evaluation Criteria in Solid Tumors (RECIST) version 1.1 is the most commonly used criteria to assess tumor response [1]. The RECIST criteria use the uni-dimensional measurement [1, 2], instead of the bi-dimensional criterion in the WHO guidelines [3]. The objective tumor response is decided by comparing the interval change in the tumor size. However, morphological measurement based on computed tomography (CT) has limitations in tumors with obscure margins, cystic lesion, or scar tissue. Especially, the measurements of the longest diameter of lesions on CT in patients with gastrointestinal tumors are not always possible. There is also concern about assessing tumor response with the RECIST 1.1 in patients receiving novel targeted agents [4]. When the RECIST 1.1 was revised, patients treated with targeted agents were not included in the data warehouse [5]. Targeted agents are usually more cytostatic than cytocidal, acting as signal transduction inhibitors. They tend to induce necrosis and cystic change in solid tumors without necessarily producing tumor shrinkage [6]. Therefore, anatomic imaging alone may have major limitations, particularly in assessing the activity of targeted agents that stabilize diseases. With increasing use of targeted agents, new evaluation methods were needed to accurately monitor tumor response.

Positron emission tomography (PET) with ^18^F-fluorodeoxyglucose (FDG) has been widely adopted as a tool for evaluating metabolic activity in tumors. FDG PET is also increasingly performed to detect earlier tumor responses to anti-cancer therapies [7]. It can allow the measurement of tumor response even in the absence of anatomic changes. Recently the PET Response Criteria in Solid Tumors (PERCIST) was proposed as a new method for the quantitative assessment of metabolic changes in solid tumors [8]. It has been shown to correlate well with anatomic response and even survival in some cases [9–11]. The PERCIST may provide clinicians with more accurate information of therapeutic response at earlier stage of treatment. However, the assessments of tumor responses have shown considerable difference between the PERCIST and RECIST criteria in several studies [11–15]. Therefore, the advantage of the PERCIST over the RECIST criteria need to be further evaluated. This pooled study was conducted to compare tumor response assessment according to the anatomic (RECIST) and metabolic (PERCIST) criteria in patients with malignant solid tumors.

## RESULTS

### Eligible studies

There were seven articles [11–17] in the literature comparing tumor response by the anatomic (RECIST 1.0, RECIST 1.1, or modified RECIST 1.1) and metabolic (PERCIST) criteria in patients with solid tumors. However, one article that compared the RECIST 1.1 and PERCIST in advanced non-small cell lung cancer (NSCLC) had no details of response classification [17]. Finally, six studies [11–16] including data on the comparison of the RECIST and PERCIST criteria were selected.

One article [12] compared tumor responses to chemotherapy in NSCLC using the PERCIST and modified RECIST 1.1 (mRECIST 1.1) in which only one single largest target lesion is measured. Two eligible articles [11, 13] compared tumor response using the RECIST 1.0 and PERCIST, and the remaining three included the data comparing the RECIST 1.1 and PERCIST in a variety of solid tumors [14–16].

### Patients' characteristics

A total of 273 patients with various solid tumors were collected from the six articles. However, 5 patients from the study with esophageal cancer [16] were excluded from this analysis because their diseases were not classifiable according to the RECIST 1.1. Finally 268 patients were included in this pooled study; 81 with colorectal cancer [13, 14], 60 with lung cancer [12, 14], 48 with esophageal cancer [15, 16], 28 with breast cancer [14, 16], 14 with basal cell carcinoma [11], 12 with stomach cancer [14], 10 with head and neck cancer [14, 16], 5 with primitive neuroectodermal tumor (PNET) [16], and 11 with other rare types of cancers [16] (Table 1). Except for 46 patients with esophageal cancer who received neoadjuvant chemotherapy [15], 222 patients (82.8%) were treated in palliative setting. Fourteen patients with basal cell carcinoma received vismodegib, the first Hedgehog signaling pathway targeting agent [11].

**Table 1:** Summary of six studies comparing the RECIST and PERCIST criteria

Reference	Tumor type	No. of pts	Treatment	Comparison	Discordant rate	Details of discordance	Correlation of response criteria and survival
						RECIST → PERCIST	
Thacker *et al.* [11]	Basal cell carcinoma	14	Targeted agent (Vismodegib)	RECIST 1.0 vs. PERCIST	50% (7/14)	2 PR → 1 CMR	The PERCIST was associated with PFS and OS.
						1 SMD	
						4 SD → 4 PMR	
						1 PD → 1 SMD	
Ding al *et al*. [12]	Non-small cell lung cancer	44	Palliative chemotherapy	mRECIST 1.1 vs. PERCIST	34.1% (15/44)	6 PR → 4 CMR	Only the PERCIST was a significant prognostic factors for DFS (HR = 3.20, *P* < 0.001)
						2 SMD	
						9 SD → 1 CMR	
						7 PMR	
						1 PMD	
Skougaard *et al.* [13]	Colorectal cancer	61	Palliative chemotherapy	RECIST 1.0 vs. PERCIST	54.1% (33/61)	1 PR → 1 SMD	Survival was not different between PR and SD (median OS 21. 4 vs. 12.2 months, *P* = 0.082). Survival was different between PMR and SMD (median OS 14.5 vs. 6.9 months, *P* < 0.0005).
						24 SD → 20 PMR	
						4 PMD	
						8 PD → 4 PMR	
						4 SMD	
Aras et al. [14]	Colorectal cancer	20	Palliative chemotherapy	RECIST 1.1 Vs. PERCIST	18.3% (11/60)	4 PR → 3 CMR	Not available
	Lung cancer	16				1 SMD	
	Stomach cancer	12				7 SD → 7 PMR	
	Head & neck cancer	6					
	Breast cancer	6					
Yanagawa *et al*.*[15]	Esophageal cancer	46	Neoadjuvant chemotherapy	RECIST 1.1 vs. PERCIST	56.5% (26/46)	13 PR → 13 CMR	Only the PERCIST was a significant prognostic factors for DFS (HR = 4.060, *P* < 0.001) and OS (HR = 8.953, *P* = 0.034)
						13 SD → 3 CMR	
						10 PMR	
Agrawal *et al*. [16]	Breast cancer	22	Metronomic palliative chemotherapy	RECIST 1.1 vs. PERCIST	20.6% (9/43)	8 SD → 1 CMR	Not available
	PNET	5				1 PMR	
	Head & neck cancer	4				6 PMD	
	Sarcoma	3					
	NHL	2					
	Esophageal cancer	2				1 PD → 1 CMR	
	Gall bladder cancer	1					
	Ovarian cancer	1					
	Paraganglioma	1					
	Hemangiopericytoma	1					
	Ganglioneuroblastoma	1					
Summary	Colorectal cancer	81		RECIST vs PERCIST	37.7% (101/268)	26 PR → 21 CMR	SD by the RECIST was most frequently shifted by the PERCIST (65/101).
	Lung cancer	60				5 SMD	
	Esophageal cancer	48				65 SD → 5 CMR	The PERCIST was an independent prognostic factor for survival.
	Breast cancer	28				49 PMR	
	Basal cell carcinoma	14				11 PMD	
	Stomach cancer	12					
	Head & neck cancer	10					
	PNET	5				10 PD → 1 CMR	
	Sarcoma	3				4 PMR	
	NHL	2				5 SMD	
	Others	6					

Abbreviations: CMR, complete metabolic response; CR, complete response; DFS, disease free survival; HR, hazard ration; NHL, non-Hodgkin's lymphoma; OS, overall survival; PD, progressive disease; PERCIST, PET Response Criteria in Solid Tumors; PFS, progression-free survival; PMD, progressive metabolic disease; PMR, partial metabolic response; PNET, primitive neuroectodermal tumor; PR, partial response; RECIST, Response Evaluation Criteria in Solid Tumors; SD, stable disease; SMD, stable metabolic disease.

* Five patients were excluded from this analysis because their disease was not classifiable according to the RECIST 1.1

### Tumor responses

Because the RECIST 1.1 includes PET scans for the detection of new lesions, we redefined the tumor response of two patients (one with PNET and the other with ganlioneuroblastoma) with a new hypermetabolic marrow lesion detected on FDG-PET as progressive disease (PD) [15]. Then, we compared tumor responses between the RECIST (RECIST 1.0, RECIST 1.1, and mRECIST 1.1) and PERCIST (Table 1 and 2). The agreement of tumor response between two criteria was moderate (linear weighted k = 0.590, 95% confidence interval, 0.523-0.657). Of 268 patients, 101 (37.7%) showed discordance in the assessment of tumor responses between two criteria: When adopting the PERCIST, tumor response was upgraded in 85 patients and downgraded in 16. The details of the patients showing disagreement between the two criteria were described in Table 1. Of 65 patients with SD, 54 were upgraded to complete metabolic response (CMR) (5) or partial metabolic response (PMR) (49) and 11 were downgraded to progressive metabolic disease (PMD) by the PERCIST. Of 26 patients with partial response (PR), 21 were reclassified as CMR and 5 as stable metabolic response (SMD). The overall response rates (ORRs), which were estimated in total regardless of the primary tumor sites, were significantly different between two criteria (35.1% by the RECIST vs. 54.1% by the PERCIST, *P* < 0.0001).

**Table 2:** Comparison of tumor responses according to the RECIST (RECIST 1.0, RECIST 1.1, and modified RECIST 1.1) and PERCIST criteria

Tumor response by RECIST	Tumor response by PERCIST				Total
	CMR	PMR	SMD	PMD	
CR	5	0	0	0	5
PR	21	60	5	0	86
SD	5	49	46	11	111
PD	1	4	5	56	66
Total	32	113	56	67	268

Abbreviations: CMR, complete metabolic response; CR, complete response; PMD, progressive metabolic disease; PD, progressive disease; PERCIST, PET Response Criteria in Solid Tumors; PMR, partial metabolic response; PR, partial response; RECIST, Response Evaluation Criteria in Solid Tumors; SD, stable disease; SMD, stable metabolic disease.

The level of concordance of tumor responses between the RECIST and PERCIST 1.0 is 0.590 (liner weighted k, with 95% confidence interval, 0.523-0.657).

The overall response rates were significantly different between two criteria (35.1% by the RECIST vs. 54.1% by the PERCIST, *P* < 0.0001).

When we compared tumor responses between the RECIST 1.1 and PERCIST criteria in 149 patients who were assessed by the RECIST 1.1, the level of agreement (k = 0.689) and the ORRs (33.6% vs. 48.3%, *P* = 0.010) showed similar results (Table 3).

**Table 3:** Comparison of tumor responses according to the RECIST version 1.1 and PERCIST criteria

Tumor response by RECIST 1.1	Tumor response by PERCIST				Total
	CMR	PMR	SMD	PMD	
CR	3	0	0	0	3
PR	16	30	1	0	47
SD	4	18	23	6	51
PD	1	0	0	47	48
Total	24	48	24	53	149

Abbreviations: CMR, complete metabolic response; CR, complete response; PMD, progressive metabolic disease; PD, progressive disease; PERCIST, PET Response Criteria in Solid Tumors; PMR, partial metabolic response; PR, partial response; RECIST, Response Evaluation Criteria in Solid Tumors; SD, stable disease; SMD, stable metabolic disease.

The level of concordance of tumor responses between the RECISI 1.1 and PERCIST is 0.689 (liner weighted k, with 95% confidence interval, 0.612-0.763).

The overall response rates were significantly different between two criteria (33.6% by the RECIST vs. 48.3% by the PERCIST, *P* = 0.010)

## DISCUSSION

In this pooled analysis, we investigated the impact of the PERCIST on the assessment of tumor response in patients with solid tumors. There was a considerable disagreement in the assessment of tumor response between the RECIST and PERCIST. Compared to the RECIST criteria, the PERCIST increased significantly overall tumor response rate.

The RECIST 1.1 includes ^18^F-FDG PET scans for the detection of new lesions [1]. It may be useful for early detecting bone marrow involvement of cancers [16, 18]. PET is also increasingly adopted to monitor tumor responses to anti-cancer therapies in solid tumors [9, 10, 19, 20]. The PERCIST is a new standardized method to quantitatively assess metabolic tumor response with PET. It was proposed after reviewing approximately 3,000 relevant references about qualitative and quantitative assessment of tumor response with ^18^F-FDG PET [8]. The PERCIST has shown various level of concordance with the RECIST criteria in the assessment of tumor responses in six studies with a small number of patients [11–16]. Thus, we conducted this pooled analysis to compare tumor response between anatomic (RECIST) and metabolic (PERCIST) criteria. Because the RECIST 1.1 showed high concordance with the RECIST 1.0 or mRECIST 1.1 in the assessment of tumor response [21–23], we included three versions of the RECIST criteria (RECIST 1.0, RECIST 1.1, and mRECIST 1.1) in the study.

In this pooled analysis using the six studies, the agreement of tumor response between the RECIST and PERCIST was just moderate (k = 0.590). Of 268 patients, 101 (37.7%) showed discordance in the assessment of tumor responses between two criteria. The PERCIST upgraded tumor response in 85 patients (84.2%) and downgraded in 16 (15.8%). As a result, the ORR was significantly increased when adopting the PERCIST instead of the RECIST criteria (35.1 vs. 54.1%, *P* < 0.0001). The shift of tumor response occurred most frequently in patients with SD by the RECIST criteria. Of 65 patients with SD, 11 were downgraded to PMD by the PERCIST. The PERCIST upgraded 10 patients with PD to CMR (1), PMR (4), or SMD (5). Patients showing PD need to change therapeutic regimen in clinical practice. If the PERCIST had been adopted for the assessment of tumor response instead of the RECIST criteria, it would have changed therapeutic plan in 7.8% (21/268) of the study patients. Therefore, these findings indicate that the clinical impact of the PERCIST on making therapeutic decisions may be significant.

Early prediction of treatment response is of great value to avoid unnecessary toxicity and cost of ineffective treatment. Anatomic responses based on the size of tumor may lag weeks or months behind metabolic response [24]. The PERCIST may offer clinicians more accurate information of therapeutic response at earlier stage of treatment. PET can detect metabolic changes after chemotherapy even when there are no or minimal morphological changes [10, 20], which may explain the reason why tumor responses were upgraded by the PERCIST in 54 patients with SD by the RECIST. The PERCIST was an independent prognostic factor for survival [12, 13, 15, 17], while the RECIST lost its prognostic value in multivariate analysis [12, 15]. Thus, the PERCIST might be more suitable for assessing tumor response to anti-cancer treatment than the RECIST criteria.

This pooled study has several limitations needed to be noted. First, as mentioned earlier, we included three versions of the RECIST criteria (RECIST 1.0, RECIST 1.1, and mRECIST 1.1) in this pooled study. However, when we compared tumor responses only between the RECIST 1.1 and PERCIST, the level of agreement showed the similar results (Table 3). Second, the data included in this study were quite heterogeneous with different types of tumor and different kinds of chemotherapy. Therefore, it is necessary to verify the results in studies with larger homogeneous patients' cohort. Third, this study could not compare the prognostic role of the RECIST and PERCIST. Two articles had no information regarding survival [14, 16]. Although the PERCIST was a potential predictor of outcomes in four studies [11–13, 15], survival data were not enough to compare prognostic value of two criteria.

In conclusion, this pooled analysis demonstrates that the concordance of tumor responses between the RECIST and PERCIST is not excellent. When adopting the PERCIST instead of the RECIST criteria, the ORR was significantly increased. Although the PERCIST seems to be a potential predictor of outcomes, its prognostic value needs to be investigated in studies with larger homogeneous patients' cohort.

## MATERILAS AND METHODS

### Searching strategy

We thoroughly looked into all potentially relevant studies written in English through the following searching strategy. A systematic literature search of MEDLINE, PUBMED, EMBASE, and Google scholar from 2009 when the PERCIST criteria were proposed to January 2016 was performed to identify articles including the following terms in their titles, abstracts, or keywords; ‘tumor response’, ‘RECIST’, or ‘PERCIST’. In addition, we searched all the references of identified relevant articles and reviews. We used the ‘related articles' feature in the PUBMED to identify the related articles.

### Study selection criteria

Articles were considered for inclusion in this pooled study if they assessed tumor response by the RECIST or PERCIST. The used RECIST criteria included RECIST version 1.0, RECIST version 1.1, and mRECIST 1.1. The searched articles were screened again by full text review, and the original articles which compared the assessment of tumor response according to the RECIST and PERCIST were included in the study.

### Definition of tumor responses

The objective tumor response according to the RECIST criteria in each study were defined as follows [1, 2]: *(i)* Complete response: disappearance of all lesions; *(ii)* PR: at least a 30% decrease in the sum of diameters of target lesions and no new lesion; *(iii)* PD: more than 20% increase in the sum of diameters of target lesions (and also an absolute increase of at least 5 mm in the RECIST 1.1) or the appearance of new lesion on CT (or PET in the RECIST 1.1); *(iv)* SD: neither sufficient shrinkage to qualify PR nor sufficient increase to qualify for PD.

The metabolic tumor response according to the PERCIST were defined as follows [9]: *(i)* CMR: complete resolution of ^18^F-FDG uptake; *(ii)* PMR: a minimum of 30% reduction of the peak lean body mass standardized uptake value (SULpeak) in the target volume; *(iii)* PMD: more than 30% increase in the SULpeak of the FDG uptake or appearance of FDG avid new lesions; *(iv)* SMD: not qualify for CMR, PMR or PMD.

### Statistical analyses

Chi-square test was used to compare the ORRs between two groups. P-values less than 0.05 were considered significant. The level of concordance in tumor responses between two criteria was calculated using linear weighted kappa statistics. Agreement between the two criteria was interpreted as poor (k < 0), slight (k = 0 – 0.20), fair (k = 0.21 – 0.40), moderate (k = 0.41 – 0.60), substantial (k = 0.61 – 0.80), and almost perfect (k > 0.80).
